# Extraction and selection of high-molecular-weight DNA for long-read sequencing from *Chlamydomonas reinhardtii*

**DOI:** 10.1371/journal.pone.0297014

**Published:** 2024-02-08

**Authors:** Frédéric Chaux, Nicolas Agier, Stephan Eberhard, Zhou Xu

**Affiliations:** 1 CNRS, UMR7238, Institut de Biologie Paris‐Seine, Laboratory of Computational and Quantitative Biology, Sorbonne Université, Paris, France; 2 CNRS, UMR7141, Institut de Biologie Physico-Chimique, Laboratory of Chloroplast Biology and Light-Sensing in Microalgae, Sorbonne Université, Paris, France; Sathyabama Institute of Science and Technology, INDIA

## Abstract

Recent advances in long-read sequencing technologies have enabled the complete assembly of eukaryotic genomes from telomere to telomere by allowing repeated regions to be fully sequenced and assembled, thus filling the gaps left by previous short-read sequencing methods. Furthermore, long-read sequencing can also help characterizing structural variants, with applications in the fields of genome evolution or cancer genomics. For many organisms, the main bottleneck to sequence long reads remains the lack of robust methods to obtain high-molecular-weight (HMW) DNA. For this purpose, we developed an optimized protocol to extract DNA suitable for long-read sequencing from the unicellular green alga *Chlamydomonas reinhardtii*, based on CTAB/phenol extraction followed by a size selection step for long DNA molecules. We provide validation results for the extraction protocol, as well as statistics obtained with Oxford Nanopore Technologies sequencing.

## Introduction

In recent years, long-read sequencing technologies, such as the ones developed by Pacific Biosciences (PacBio) and Oxford Nanopore Technologies (Nanopore), have emerged as a solution to the pitfalls of short-read technologies in the detection of structural variants and in assembling repeated sequences and other complex regions [1]. Additionally, because native DNA is used, long-read technologies can directly detect a variety of modified bases, including the most commonly studied methylated cytosines [2, 3]. For their applications in genome assembly and structural variant detection, these technologies typically sequence DNA molecules ranging in size from kilobases to hundreds of kilobases as a continuous read. Reads traversing repeated sequences are necessary to correctly assemble neighboring regions, with longer reads enabling more contiguous genome assemblies. Today, the major bottleneck to sequence long reads comes from the ability to extract high-quality DNA devoid of polyphenol and polysaccharide contaminants with sizes compatible with this purpose. This is especially true for most plant tissues and algae cells, because polyphenols and polysaccharides are often co-extracted with DNA and can inhibit downstream applications such as sequencing [4, 5].

*Chlamydomonas reinhardtii* is a unicellular green alga that is widely used as a model organism to study photosynthesis and cellular motility [6], and is an organism of choice for biotechnological application, with many synthetic biology tools being currently developed [7, 8]. In *C*. *reinhardtii*, as for other plants and algae, contending with phenolic and polysaccharide contaminants while preserving HMW DNA is a major challenge and requires an optimized protocol. PacBio and Nanopore sequencing have been performed on this organism, contributing to important advances in our understanding of its genome structure and content, base modifications and evolution [9–16]. However, it appears that a size selection step can substantially enrich for longer molecules, as noted in [14, 15] and as we demonstrate in this work. An efficient and well documented protocol is therefore needed for sequencing projects that require long DNA molecules.

Here, we present a detailed protocol dedicated to efficiently extract and select HMW DNA from *C*. *reinhardtii* cells. The protocol minimizes DNA-shearing manipulations [17] and comprises an additional step to enrich for HMW DNA. We validated the method by pulse-field gel electrophoresis (PFGE) and measurement of read length from Nanopore sequencing.

## Materials and methods

The protocol described in this peer-reviewed article is published on protocols.io, dx.doi.org/10.17504/protocols.io.8epv59j9jg1b/v2 and is included for printing purposes as S1 File.

### Nanopore sequencing

Sequencing libraries were prepared as per manufacturer’s recommendations, using NEBNext companion module (E7180S, NEB) and Ligation Sequencing Kit SQK LSK-109 (Nanoporetech), except for the ligation time, which we increased to 30 min. For each run, 500 ng were loaded on MinION flow cells (R9.4.1, Nanoporetech) and sequenced for 6h to 16h, depending on flow-cell kinetics. Libraries were loaded at least twice, with 1h wash using the manufacturer’s washing buffer (EXP-WSH004) between runs. Basecalling was performed using Guppy (version 4.3.4) with parameters set to “high accuracy”.

## Results

We extracted genomic DNA following the presented protocol (S1 File) and applied size selection using the Short Read Eliminator (SRE) kit (Circulomics), an easy-to-use method that does not require dedicated devices which is based on a length-dependent precipitation of nucleic acids driven by polyvinylpyrrolidone crowding. Large amounts of small DNA fragments can be detrimental for long-read Nanopore sequencing [18], not only because the subsequent reads are short, but also because these molecules can outcompete the longer ones, both for adapter ligation and pore usage, thus yielding suboptimal results.

**Fig 1:**
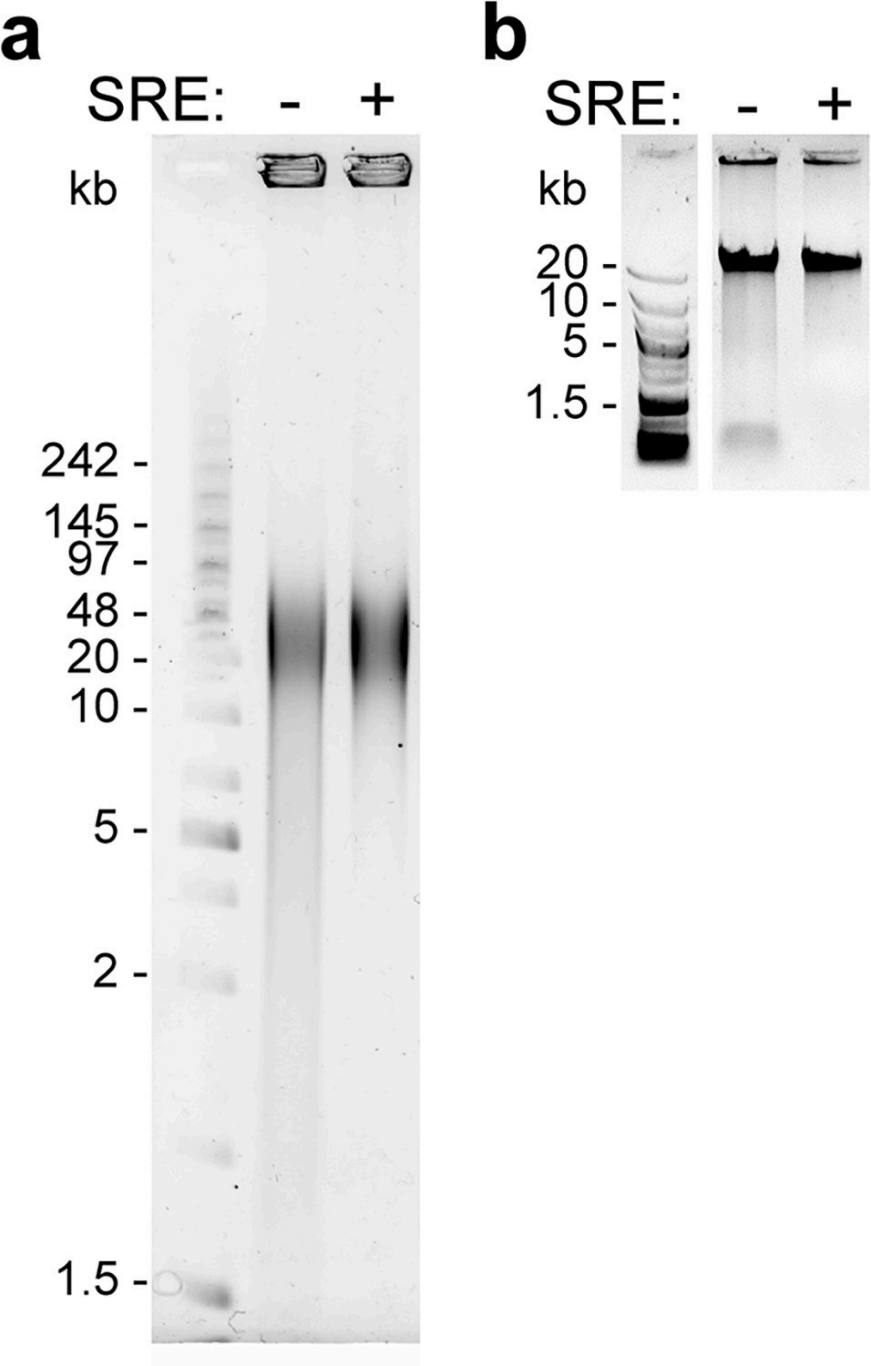
Visualization of extracted genomic DNA size distributions. (a) PFGE using 0.5 μg of DNA prepared with (+) or without (-) SRE size-selection, embedded in 30 μl of 0.5% low-melting agarose plugs, migrated in a 1% SeaKem GTG agarose (Lonza) gel. The ladder is a mix of PFG mid-range (N0342S, NEB) and GeneRuler 1 kb Plus (SM1331, ThermoFischer). Electrophoresis conditions: 0.5X TBE (Tris Borate EDTA) buffer, 6 V.cm^-1^, 120° angle, for 11h, switching time ramp from 1 to 60 seconds. Gel stained in ethidium bromide and imaged with UV. (b) Standard gel electrophoresis (0.3% agarose) of the indicated samples. GeneRuler 1 kb Plus (SM1331, ThermoFischer) is used as the ladder. See S3 Fig for the uncropped images.

The size distribution of the extracted DNA was assessed by PFGE and Nanopore sequencing, with and without size-selection for HMW DNA. Samples were migrated in a pulse field, stained by ethidium bromide and imaged with UV light (Fig 1A). The DNA molecules extracted without size selection migrated as a large smear spread between approximately 1.5 and 150 kb. After size selection with the SRE kit, the upper part of the distribution remained unchanged while the low-molecular-weight fragments (< 10 kb) were visibly reduced. We made a similar observation after electrophoresis and staining of the samples in a 0.3% agarose gel (Fig 1B).

**Fig 2:**
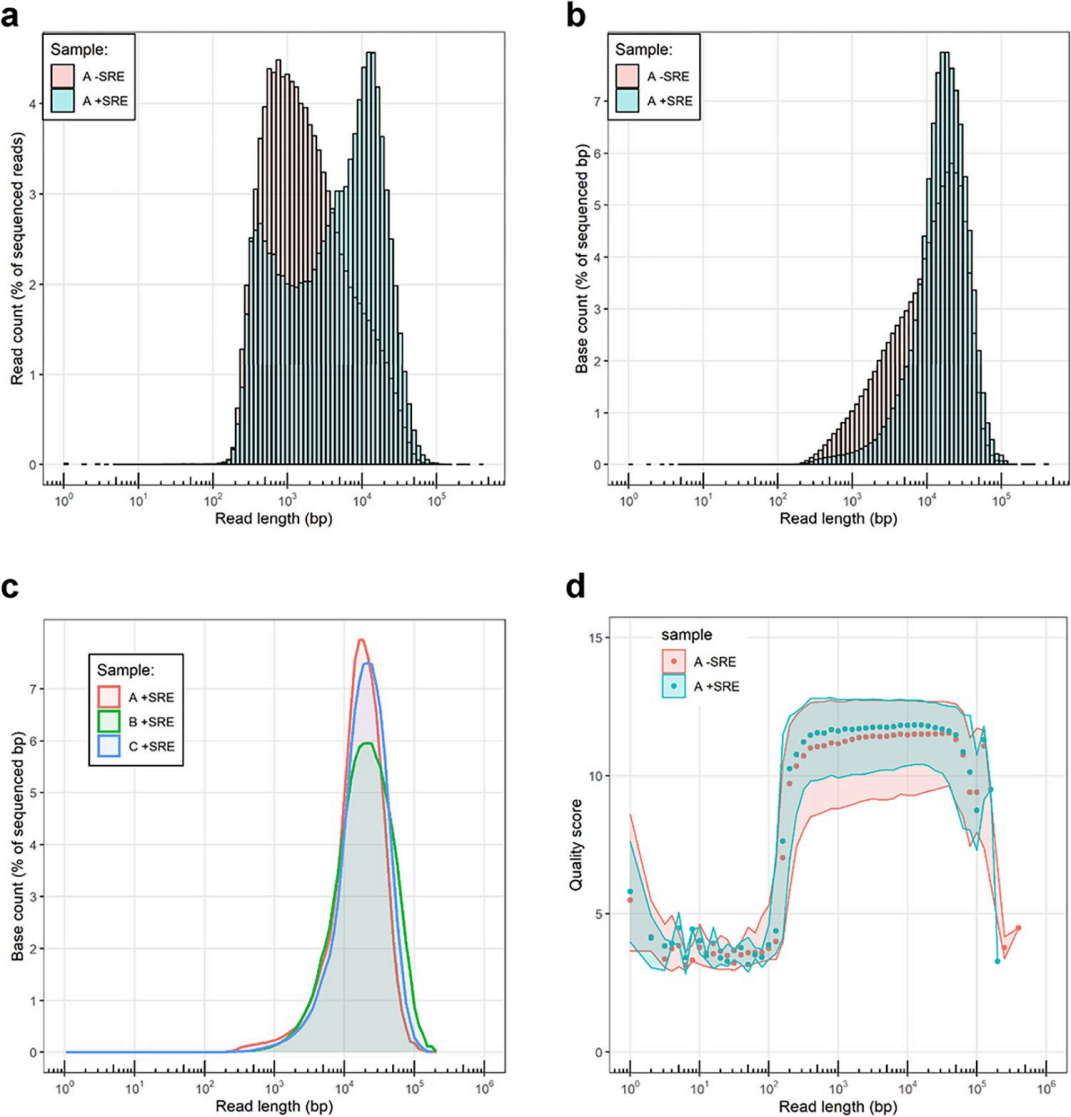
Distributions of read length in Nanopore-sequenced datasets. (a, b) Count percentage of (a) reads and of (b) bases as a function of read length obtained from genomic DNA of *C*. *reinhardtii* (experiment “A”, see S1 Table) with or without size selection (+SRE and -SRE). (c) Count of bases after size-selection (+SRE) as a function of read length obtained from three different biological samples (see S1 Table and S2 Fig). (d) Quality score for individual reads, grouped into bins of 0.1 log unit for samples “A-SRE” and “A+SRE”. The shaded areas represent the values between the 1^st^ and 3^rd^ quartiles.

Size-selection of DNA fragments before preparation of libraries for Nanopore sequencing led to a substantially decreased number of shorter molecules and an enrichment of longer ones (Fig 2A and 2B), without negatively affecting read quality (Fig 2D) and with no effect on genome-wide sequencing depth (S1 Fig). Size-selection doubled the mean read length, increased the N50 from 12 kb to 17 kb, with reads in the top decile being longer than 21 kb (S1 Table). The length distribution after size-selection was robust across different experiments using two other independent biological samples, and reached an N50 of up to 20 kb and a top decile length of up to 27 kb (Fig 2C and S1 Table). The longest molecules we sequenced were over 100 kb, which are instrumental for genome assemblies. Indeed, we recently assembled the genome of *C*. *reinhardtii* based on these reads and found a genome size between 114 and 117.7 Mb [15], depending on the assembler, which is consistent with the 114 Mb of the recently released version 6 of the reference genome [16]. Overall, this protocol and the resulting quality and length of the DNA molecules are suitable for reaching highly contiguous genome assemblies.

## Supporting information

S1 File Step-by-step protocol, also available on protocols.io: dx.doi.org/10.17504/protocols.io.8epv59j9jg1b/v2. (PDF)

S1 Table Summary statistics for 6 DNA preparations and sequencing experiments. Major limiting outputs are shown in red. ^a^ https://www.chlamylibrary.org and reference [19]. ^b^ with quality > 7. ^c^ as per manufacturer’s protocol (Monarch® HMW DNA Extraction Kit for Tissue Cat. no. T3060L, New England Biolabs). ^d^ cell lysis using DNeasy Maxi Plant (Cat. no. 68163, Qiagen) as in [20] and purification using Genomic-tip 100/G (Cat. no. 10243, Qiagen), then AMPure beads (Cat. no. A63880, Beckman Coulter).
(PDF)

S1 Fig Genome-wide sequencing depth normalized to the median, for all chromosomes, using DNA obtained with (+) or without (-) SRE size selection. (PDF)

S2 Fig Count percentage of bases as a function of read length with alternative sample preparations without size selection (-SRE). See S1 Table for details. Sample C was sequenced in the presence of control DNA (“DNA CS” from Oxford Nanopore sequencing), which peaked at 3 kb.
(PDF)

S3 Fig Raw images. (PDF)
