# Supplementary material for: Extraction and selection of high-molecular-weight DNA for long-read sequencing from Chlamydomonas reinhardtii
Source: PLoS One. 2024 Feb 8;19(2):e0297014. doi: 10.1371/journal.pone.0297014 (PMC10852265; doi:10.1371/journal.pone.0297014)
Supplement: S1 File — (PDF) [file pone.0297014.s001.pdf]

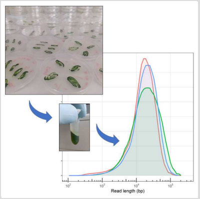

2

# Extraction and selection of high-molecular-weight DNA for long-read sequencing from *Chlamydomonas reinhardtii* V.2

Frédéric Chaux-Jukic[1], Nicolas Agier[1], Stephan Eberhard[2], Zhou Xu[1]

[1]Sorbonne Université, CNRS, UMR7238, Institut de Biologie Paris-Seine, Laboratory of Computationa l and Quantitative Biology, 75005 Paris, France;

[2]Sorbonne Université, CNRS, UMR7141, Institut de Biologie Physico-Chimique, Laboratory of Chloro plast Biology and Light-Sensing in Microalgae, 75005 Paris, France

dx.doi.org/10.17504/protocols.io.8epv59j9jg1b/v2

zhou.xu

The authors declare no conflict of interest.

Recent advances in long-read sequencing technologies have enabled the complete assembly of eukaryotic genomes from telomere to telomere by allowing repeated regions to be fully sequenced and assembled, thus filling the gaps left by previous short-read sequencing methods. Furthermore, long-read sequencing can also help characterizing structural variants, with applications in the fields of genome evolution or cancer genomics. For many organisms, the main bottleneck is to develop robust methods to obtain high-molecular-weight (HMW) DNA for whole genome sequencing purposes. We developed an optimized protocol to extract DNA suitable for long-read sequencing from the unicellular green alga *Chlamydomonas reinhardtii*, based on CTAB/phenol extraction followed by a size selection step for long DNA molecules. We provide validation results for the extraction protocol, as well as statistics obtained with Oxford Nanopore Technologies sequencing.

DOI

dx.doi.org/10.17504/protocols.io.8epv59j9jg1b/v2

Frédéric Chaux-Jukic, Nicolas Agier, Stephan Eberhard, Zhou Xu . Extraction and selection of high-molecular-weight DNA for long-read sequencing from Chlamydomonas reinhardtii . **protocols.io**
https://protocols.io/view/extraction-and-selection-of-high-molecular-weight-b9pir5ke
zhou.xu

high-molecular weight DNA, long-read sequencing, size selection, algae DNA extraction

protocol ,

Frédéric Chaux-Jukic

May 20, 2022

May 20, 2022

62922

- This protocol aims at extracting high-molecular-weight DNA molecules with maximal removal of contaminants. Both parameters are indeed crucial to take full advantage of long-read sequencing.
- This protocol was developed for the microalga *Chlamydomonas reinhardtii*, but might be adapted to other algae and land plants.
- This protocol is relatively simple and short, however, for basic molecular biology (PCR, cloning of small fragments, etc.), simpler protocols may be preferred (e.g. www.chlamycollection.org/methods/).
- The extraction steps rely mostly on home-made solutions. Purification and size selection uses commercial reagents, the composition of which is unknown to us.

- Equipment for *C. reinhardtii* culture
- Growth media: https://www.chlamycollection.org/methods/media-recipes/tap-and-tris-minimal/
- Ice bucket
- Water bath
- 2-ml tubes
- Wide-bore tips (or cut out the extremity of regular tips)
- 1.5-ml Lo-Bind tubes (Eppendorf)
- SpeedVac (optional)
- Magnetic rack

- Ultrapure water
- CTAB solution:

50 mM Tris-HCl pH 8
20 mM EDTA
1.4 M NaCl
2% CTAB (Hexadecyltrimethylammonium bromide; Sigma-Aldrich)
1% PVP 40.000 (Polyvinylpyrrolidone; Sigma-Aldrich)

- Proteinase K: prepare stock solution at 20 $mg.mL^{-1}$
- RNase A: prepare stock solution at 100 $mg.mL^{-1}$
- Phenol:Chloroform:Isoamyl alcohol (25:24:1)
- Chloroform:Isoamyl alcohol (24:1)
- Isopropanol
- Ethanol: prepare at 70% v/v in ultrapure water

- AMPure XP beads (A63880, Beckman Coulter)
- Short Read Eliminator (SRE) kit (Circulomics)

- Optional: Qubit fluorimeter, Nanodrop
- Optional: pulsed-field gel electrophoresis (PFGE) system

Wear gloves at all steps of the protocol and apply standard safety procedure for biochemistry.
Handle phenol chloroform isoamyl alcohol and chloroform isoamyl alcohol under a chemical hood.

:

The authors declare no conflict of interest.

On the day of extraction:
- Set water bath to 65°C (large enough for one 50-ml tube per sample)
- Chill isopropanol to -20°C (around 2.5 ml per sample)

## Harvesting and storage of cells

1 Grow *Chlamydomonas reinhardtii* cells in **100 mL** TAP medium under low light (~5 µmol photon.$m^{-2}.s^{-1}$) with constant shaking at **100 rpm, 25°C** , or in other conditions as

appropriate for the specific experiment.

2 Harvest the cells at the end of exponential growth phase (~$10^7$ cells.$mL^{-1}$) in 50 mL tubes by centrifugation **4000 x g, Room temperature, 00:05:00** . Discard supernatant. 5m

3 Store cell pellets at **-20 °C** (optional).

DNA extraction

4 Thaw cell pellets at **Room temperature** (not applicable if the pellets were not frozen) and put on ice.

5 Centrifuge at **4000 x g, 4°C, 00:05:00** and discard any liquid left. 5m

6 Resuspend by gentle pipetting in **3 mL** of CTAB solution preheated at **65 °C** .

> To reduce DNA shearing, from this step forward, use wide bore tips (or cut standard tips) and pipet as slowly as possible, mix gently by inverting when needed and do not vortex.

7 Add **5 µL** of proteinase K (stock solution: 20 mg.$mL^{-1}$) and **5 µL** of RNase A (stock solution: 100 mg.$mL^{-1}$), mix by pipetting and incubate at **65 °C** for **00:20:00** in a water bath. 20m

8 Centrifuge at **4000 x g, Room temperature, 00:05:00** and distribute 3 x 1 mL of each supernatant into 2 mL tubes. Discard pellet. 5m

> Efficient lysis produces a pale green pellet, of much lighter hue than the supernatant.

9 Add **1 mL** of phenol:chloroform:isoamyl alcool (25:24:1) to each tube, mix gently by 5m

inverting 10 times and centrifuge at **20000 x g, 4°C, 00:05:00** . Transfer the aqueous (upper) phase to new 2 mL tubes.

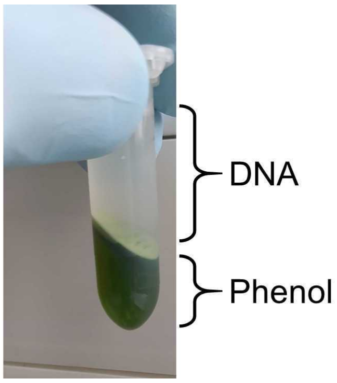


After centrifugation, the aqueous phase should be colorless, the phenol (lower) phase should be dark green and a white precipitate may form at the interface.

> When pipetting the upper phase, avoid contact with the white precipitate that usually forms at the interface. To avoid traces of phenol, do not attempt to transfer more than three quarters of the aqueous phase.

10 Add **1 µL** of proteinase K and **1 µL** of RNase A, mix gently by inverting 3 times and incubate at **50 °C** for **00:20:00** . 20m

> This step aims at completing RNA degradation and removal of DNA-binding proteins.

11 Add **1 mL** of chloroform:isoamyl alcohol (24:1) to each tube, mix gently by inverting 3 times and centrifuge at **20000 x g, 4°C, 00:05:00** . Transfer the upper phase to new 2 mL tubes. 5m

12 Add **700 µL** of isopropanol to each tube and mix gently by inverting 10-12 times. A visible DNA precipitates should form.

> If greenish/brownish contaminants precipitate together with DNA, do not use it for sequencing.

13 Centrifuge at 20000 x g, 4°C, 00:05:00 , discard the supernatant carefully, add 500 µL of ice-cold 70% ethanol without disturbing the pellet and centrifuge at 20000 x g, 4°C, 00:05:00 . 10m

14 Discard the ethanol carefully by pipetting and eliminate the remaining traces using a SpeedVac for 00:05:00 at Room temperature or leave tubes open for 00:30:00 at Room temperature . 35m

15 Resuspend the pellet in 30 µL of ultrapure water overnight at Room temperature .

## Purification and selection of high-molecular-weight DNA

16 Pool the samples corresponding to the same initial culture in one 1.5ml Lo-Bind tube, add an equal volume (e.g. 3 x 30 µL ) of AMPure XP beads, mix gently by hand for 00:05:00 . 5m

> The beads solution is relatively viscous, let it reach Room temperature , then resuspend by vortexing for 00:00:10 before pipetting from the stock and ensure complete mixing with the DNA sample.

17 Place the tubes on a magnetic rack and let the beads aggregate on the side of the tube for 00:05:00 . Discard the supernatant. Remove the tubes from the magnetic rack, resuspend the beads in 60 µL of ultrapure water for at least 00:15:00 at Room temperature . 20m

18 Place the tubes on a magnetic rack and let the beads aggregate on the side of the tube for 00:05:00 . Transfer the supernatant to new 1.5 mL Lo-Bind tubes and discard the beads. 5m

19 Measure DNA concentration and purity using a Nanodrop and/or a Qubit device.

20 Adjust each sample to ~150 ng.$\mu L^{-1}$ in 60 µL of ultra-pure water, gently mix with an equal volume of Short Read Eliminator (SRE) buffer (SRE kit, Circulomics) and centrifuge at 30m

10000 x g, 4°C, 00:30:00 . Pipet out the supernatant very carefully as the pellet is colorless and can easily be lost.

> Manufacturer's recommendation is to measure input DNA concentration using Qubit (Short Read Eliminator Kit - Handbook v2.0)

21 Gently add 200 µL of freshly prepared 70% ethanol to the pellet. Wash by centrifuging at 10000 x g, 4°C, 00:02:00 and discarding the ethanol. 2m

22 Repeat Step 21.

23 Air dry the pellet for 00:10:00 at Room temperature and resuspend in 50 µL of pre-warmed EB buffer (SRE kit, Circulomics) by incubating at 50 °C for 00:20:00 . 30m

24 Proceed to long-read sequencing or store at 4 °C for later use (at least several weeks). Do not freeze to avoid DNA shearing.

Quality check

25 DNA purity can be assessed by Qubit or Nanodrop.

26 Absence of small fragments can be assessed by electrophoresis on agarose gel and size distribution can be assessed by pulsed-field gel electrophoresis (PFGE).
