# Supplementary material for: Extraction and selection of high-molecular-weight DNA for long-read sequencing from Chlamydomonas reinhardtii
Source: PLoS One. 2024 Feb 8;19(2):e0297014. doi: 10.1371/journal.pone.0297014 (PMC10852265; doi:10.1371/journal.pone.0297014)
Supplement: S1 Table — Major limiting outputs are shown in red. a https://www.chlamylibrary.org and reference [19]. b with quality > 7. c as per manufacturer’s protocol (Monarch® HMW DNA Extraction Kit for Tissue Cat. no. T3060L, New England Biolabs). d cell lysis using DNeasy Maxi Plant (Cat. no. 68163, Qiagen) as in [20] and purification using Genomic-tip 100/G (Cat. no. 10243, Qiagen), then AMPure beads (Cat. no. A63880, Beckman Coulter). (PDF) [file pone.0297014.s002.pdf]

S2 Table

Sample	CLiP library [a]	DNA extraction	Library preparation	Read count	Base count (Gb)	Read length quantiles (kb)			Median read length weighted by base count ("N50") (kb)	Top 3 longest reads [b] (kb)		
						Median	Q90	Q99				
A +SRE	LMJ.RY0402.077111	This protocol	LSK109 +barcodes	232 076	2,0	5,0	21	44	17	142	121	120
A -SRE		This protocol (except SRE)	LSK109	850 066	3,6	1,5	12	34	12	127	117	112
B +SRE	CC-4533 (cMJ030)	This protocol	LSK109	280 614	3,1	6,7	26	64	20	213	201	191
B -SRE		Monarch HMW DNA kit [c]	LSK109	177 794	1,1	2,5	15	53	15	199	177	175
C +SRE	LMJ.RY0402.209904	This protocol	LSK110	344 223	3,9	8,0	27	54	20	175	166	161
C -SRE		DNeasy Plant/Tip100 [d]	LSK109	81 078	0,6	3,0	22	54	21	121	119	108
