# Supplementary figures and images for: Extraction and selection of high-molecular-weight DNA for long-read sequencing from Chlamydomonas reinhardtii
Source: PLoS One. 2024 Feb 8;19(2):e0297014. doi: 10.1371/journal.pone.0297014 (PMC10852265; doi:10.1371/journal.pone.0297014)

S3 Supplementary Figure

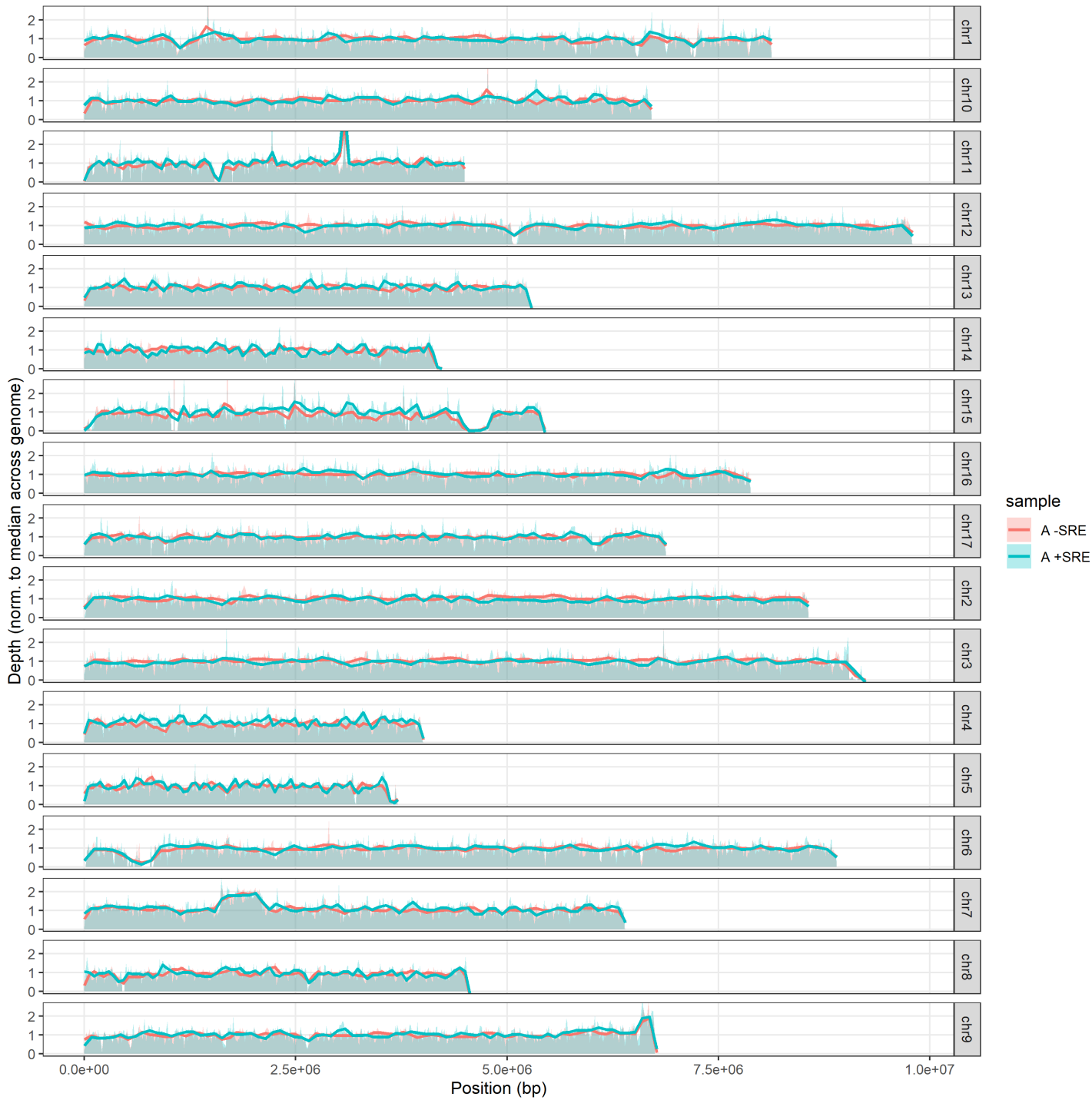

Supplement: S1 Fig — (PDF) [file pone.0297014.s003.pdf]

S4 Supplementary Figure

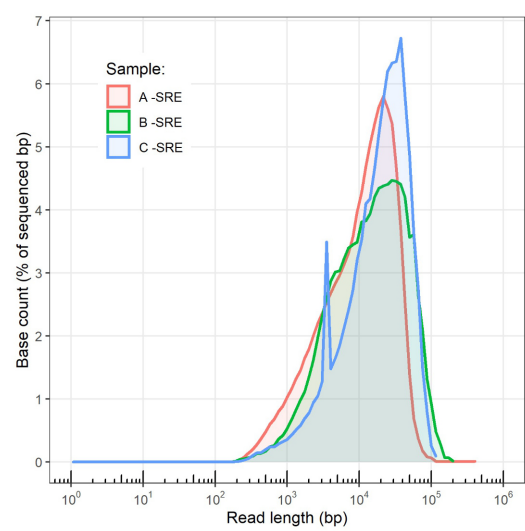

Supplement: S2 Fig — See S1 Table for details. Sample C was sequenced in the presence of control DNA (“DNA CS” from Oxford Nanopore sequencing), which peaked at 3 kb. (PDF) [file pone.0297014.s004.pdf]

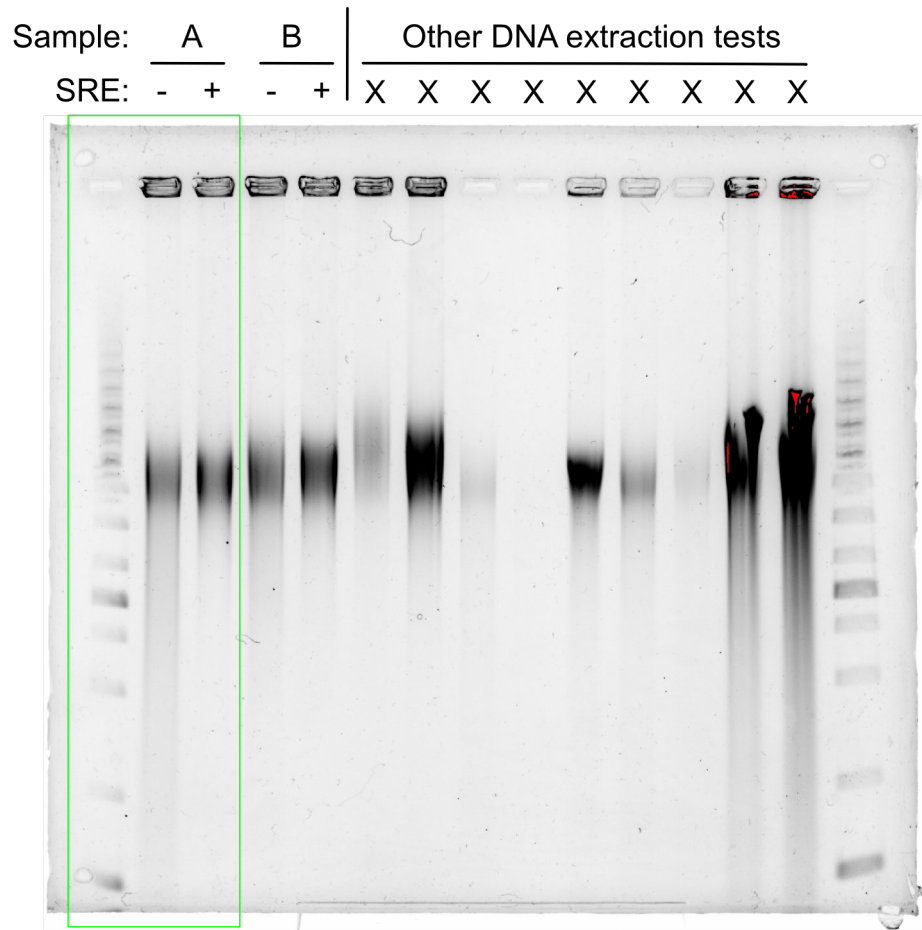

Cropped image shown in Fig. 1A

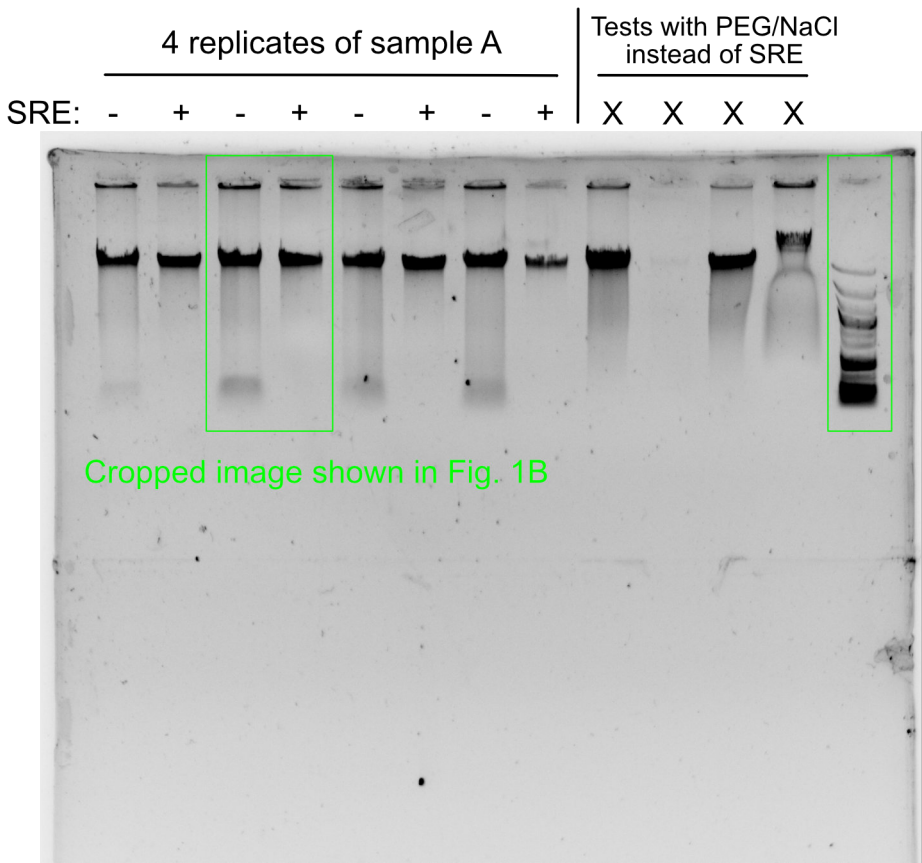

Cropped image shown in Fig. 1B

Supplement: S3 Fig — (PDF) [file pone.0297014.s005.pdf]
